# Supplementary material for: Heat Shock Transcription Factor GhHSFB2a Is Crucial for Cotton Resistance to Verticillium dahliae
Source: Int J Mol Sci. 2023 Jan 17;24(3):1845. doi: 10.3390/ijms24031845 (PMC9916287; doi:10.3390/ijms24031845)
Supplement: Supplementary file 1 [file ijms-24-01845-s001.zip › ijms-2136137-supplementary.pdf]

International Journal of *Molecular Sciences*

MDPI

Supplementary Material

Table S1. The primers used in this study[1]

Construct names	Primer names	Primer sequences (5′-3′)
pYBA1132	1132-GhHSFB2a-F	TCTAGAACTAGT**GGATCC**ATGGCTCCGCCGCCGGTGGA
	1132-GhHSFB2a-R	TAAGCTTGATATC**GAATTC**ATTACAAACCCTTTGATTCGC
pTRV2	pTRV2-GhHSFB2a-F	TCTGTGAGTAAGGTTACC**GAATTC**GGTGTTCGCTCGTGATTTGCTTC
	pTRV2-GhHSFB2a-R	ACGCGTGAGCTCGGTACC**GGATCC**ACCTCGGACTCGTTTCTTCTTCG
pCAMBIA2300-35S-OCS	2300-GhHSFB2a-F	GGACAGGGTACCCGG**GGATCC**ATGGCTCCGCCGCCGGTG
	2300-GhHSFB2a-R	CACCATGGTACTAGT**GTCGAC**ATTACAAACCCTTTGATTCGCTCG
RT-qPCR	qGhHSFB2a-F	ACGGGAACGGCGGAATCACAAAG
	qGhHSFB2a-R	AGGAAGCAAATCACGAGCGAACA
	UBQ-F	AGCTCGGATACGATTGATAACG
	UBQ-R	GAAGACGAAGAACAAGGGGAAG
	Vd-ITS-F	TCCGTAGGTGAACCTGCGG
	Vd-ITS-R	TCCTCCGCTTATTGATATGC
	AtRubisco-F	GCAAGTGTTGGGTTCAAAGCTGGTG
	AtRubisco-R	CCAGGTTGAGGAGTTACTCGGAATGCTG
	qGhNCED3-F	AATGATGCACGATTTCGCCATCA
	qGhNCED3-R	GCAATCAGGTGCCTCAACCC
	qGhNCED6-F	CCGGCGAGGGTGATCTTGA
	qGhNCED6-R	TCATTGTTGCCTGGTCGATGTC
	qGhERF114-F	GGAGATCCGAGACCCGAAACG

	qGhERF114-R	CTCTCCTTCTTCTCCACTCTGC
	qGhERF061-F	CCATCAATAGCGGCCGTCG
	qGhERF061-R	GACTGACGCAGTTGCTCGTC
	qGhLox1-F	ACAACATCCCCTTGGAGATGCT
	qGhLox1-R	AGGATCTAGGTTGCTTGTTGG
	qGhCAD1-F	GGCCCCGCTGTTATGTGCA
	qGhCAD1-R	TTCAGCGTCGGAGCTGACTAAGT
PCR	GhHSFB2a-F	ATGGCTCCGCCGCCGGTGGA
	GhHSFB2a-R	ATTACAAACCCTTTGATTCGC

1. The restriction recognition sequences of *Bam*H I (GGATCC), *Eco*R I (GAATTC) and *Sal* I (GTCGAC) within the primers are in bold and underlined. Genes included in qPCR assay are *GhHSFB2a* (LOC107904615), *UBQ* (LOC107925174), *Vd-ITS* (MT899267.1), *AtRubisco* (ATCG00490), *GhNCED3* (LOC107918579), *GhNCED6* (LOC107890583), *GhERF114* (LOC 107925292), *GhERF061* (LOC107947116), *GhLox1* (LOC107919998), *GhCAD1* (LOC107953373).
